# Convergence behavior of single-step GBLUP and SNPBLUP for different termination criteria

**DOI:** 10.1186/s12711-021-00626-1

**Published:** 2021-04-09

**Authors:** Jeremie Vandenplas, Mario P. L. Calus, Herwin Eding, Mathijs van Pelt, Rob Bergsma, Cornelis Vuik

**Affiliations:** 1 Animal Breeding and Genomics, Wageningen UR, P.O. Box 338, 6700 AH Wageningen, The Netherlands; 2 CRV BV, Wassenaarweg, 20, 6843 NW Arnhem, The Netherlands; 3 Topigs Norsvin, P.O. Box 43, 6640 AA Beuningen, The Netherlands; 4 DIAM, TU Delft, Van Mourik Broekmanweg, 6, 2628 XE Delft, The Netherlands

## Abstract

**Background:**

The preconditioned conjugate gradient (PCG) method is the current method of choice for iterative solving of genetic evaluations. The relative difference between two successive iterates and the relative residual of the system of equations are usually chosen as a termination criterion for the PCG method in animal breeding. However, our initial analyses showed that these two commonly used termination criteria may report that a PCG method applied to a single-step single nucleotide polymorphism best linear unbiased prediction (ssSNPBLUP) is not converged yet, whereas the solutions are accurate enough for practical use. Therefore, the aim of this study was to propose two termination criteria that have been (partly) developed in other fields, but are new in animal breeding, and to compare their behavior to that of the two termination criteria widely used in animal breeding for the PCG method applied to ssSNPBLUP. The convergence patterns of ssSNPBLUP were also compared to the convergence patterns of single-step genomic BLUP (ssGBLUP).

**Results:**

Building upon previous work, we propose two termination criteria that take the properties of the system of equations into account. These two termination criteria are directly related to the relative error of the iterates with respect to the true solutions. Based on pig and dairy cattle datasets, we show that the preconditioned coefficient matrices of ssSNPBLUP and ssGBLUP have similar properties when using a second-level preconditioner for ssSNPBLUP. Therefore, the PCG method applied to ssSNPBLUP and ssGBLUP converged similarly based on the relative error of the iterates with respect to the true solutions. This similar convergence behavior between ssSNPBLUP and ssGBLUP was observed for both proposed termination criteria. This was, however, not the case for the termination criterion defined as the relative residual when applied to the dairy cattle evaluations.

**Conclusion:**

Our results showed that the PCG method can converge similarly when applied to ssSNPBLUP and to ssGBLUP. The two proposed termination criteria always depicted these similar convergence behaviors, and we recommend them for comparing convergence properties of different models and for routine evaluations.

**Supplementary Information:**

The online version contains supplementary material available at 10.1186/s12711-021-00626-1.

## Background

The current method of choice for genomic evaluations is the so-called single-step genomic best linear unbiased prediction (ssGBLUP) that simultaneously analyses phenotypic and pedigree information of genotyped and non-genotyped animals with genomic information of genotyped animals [1]. The ssGBLUP model considers genomic information by combining genomic and pedigree relationships into a combined genomic-pedigree relationship matrix [2, 3]. Equivalent models that directly estimate SNP effects and that do not rely on the genomic relationship matrix ${\mathbf {G}}$, hereafter called ssSNPBLUP, were also proposed [4–6].

The solving method of choice in the last two decades for breeding value estimation models is the preconditioned conjugate gradient (PCG) method with a traditional (block-)diagonal preconditioner [7]. However, when applied to ssSNPBLUP, it results in poor convergence [8, 9]. We have previously shown that using a second-level preconditioner improves the PCG convergence rate considerably [9, 10].

An important parameter of an iterative solver is its termination criterion. A termination criterion should be defined to stop the iterative process at an appropriate level of convergence. The relative difference between two successive iterates and the relative residual of the system of equations is often chosen as a termination criterion for the PCG method in animal breeding [7, 11–13]. However, our initial analyses showed that these two commonly used termination criteria may report that a PCG method applied to ssSNPBLUP is not converged yet, whereas the solutions are accurate enough for practical use. Therefore, the aim of this study was to implement termination criteria that have been (partly) developed in other fields, but are new in animal breeding, and to compare their behaviors to that of different termination criteria that are widely used in animal breeding for the PCG method applied to ssSNPBLUP. A comparison of the convergence patterns of ssSNPBLUP and of ssGBLUP was also performed.

## Methods

### Single-step genomic evaluations

In this study, we investigate the convergence behavior of the PCG method applied to ssGBLUP [2, 14] and to the ssSNPBLUP linear equations system proposed by Gengler et al. [15] and by Liu et al. [6]. The two systems of equations can be summarized as:

$$\begin{aligned} {\mathbf {C}}_{j}{\mathbf {x}}_{j}={\mathbf {b}}_{j}, \end{aligned}$$

where *j* refers to ssGBLUP (*j*=G), or to the ssSNPBLUP proposed by Liu et al. [6] (*j*=L), ${\mathbf {C}}_{j}$ is a symmetric (semi-)definite coefficient matrix, ${\mathbf {x}}_{j}$ is the vector of solutions, and ${\mathbf {b}}_{j}$ is the right-hand side of the linear system.

For simplicity, and without loss of generality, the different matrices and vectors for ssGBLUP and ssSNPBLUP are described below for an univariate animal model.

#### ssGBLUP

For ssGBLUP [1], the vector ${\mathbf {b}}_{G}$ is equal to $${\mathbf {b}}_{G}= \left[ \begin{array}{c} {\mathbf {X}}^{'}{\mathbf {R}}^{-1}{\mathbf {y}} \\ {\mathbf {W}}^{'}_{n}{\mathbf {R}}^{-1}_{n}{\mathbf {y}}_{n} \\ {\mathbf {W}}^{'}_{g}{\mathbf {R}}^{-1}_{g}{\mathbf {y}}_{g} \\ \end{array} \right]$$ where the subscripts *g* and *n* refer to $n_{g}$ genotyped and $n_{n}$ non-genotyped animals, respectively, ${\mathbf {y}}$ is the vector of records, and the matrices ${\mathbf {X}}$, ${\mathbf {W}}_{n}$ and ${\mathbf {W}}_{g}$ are incidence matrices relating records to the corresponding effects, and the matrix $${\mathbf {R}}^{-1}=\left[ \begin{array}{cc} {\mathbf {R}}^{-1}_{n} &{} {\mathbf {0}}\\ {\mathbf {0}} &{} {\mathbf {R}}^{-1}_{g} \end{array} \right]$$ is the inverse of the residual (co)variance structure matrix.

The coefficient matrix ${\mathbf {C}}_{G}$ is equal to:

$$\begin{aligned} {\mathbf {C}}_{G}= \left[ \begin{array}{ccc} {\mathbf {X}}^{'}{\mathbf {R}}^{-1}{\mathbf {X}} &{} {\mathbf {X}}^{'}_{n}{\mathbf {R}}^{-1}_{n}{\mathbf {W}}_{n} &{} {\mathbf {X}}^{'}_{g}{\mathbf {R}}^{-1}_{g}{\mathbf {W}}_{g} \\ {\mathbf {W}}^{'}_{n}{\mathbf {R}}^{-1}_{n}{\mathbf {X}}_{n} &{} {\mathbf {W}}^{'}_{n}{\mathbf {R}}^{-1}_{n}{\mathbf {W}}_{n}+{\mathbf {A}}^{nn}\sigma _{u}^{-2} &{} {\mathbf {A}}^{ng}\sigma _{u}^{-2} \\ {\mathbf {W}}^{'}_{g}{\mathbf {R}}^{-1}_{g}{\mathbf {X}}_{g} &{} {\mathbf {A}}^{gn} \sigma _{u}^{-2} &{} {\mathbf {W}}^{'}_{g}{\mathbf {R}}^{-1}_{g}{\mathbf {W}}_{g}+ \left( {\mathbf {A}}^{gg} + {\mathbf {G}}^{-1}- {\mathbf {A}}_{gg}^{-1} \right) \sigma _{u}^{-2} \\ \end{array} \right] \end{aligned}$$

where $\sigma _{u}^{-2}$ is the inverse of the additive genetic variance, $${\mathbf {A}}^{-1} = \left[ \begin{array}{cc} {\mathbf {A}}^{nn} &{} {\mathbf {A}}^{ng} \\ {\mathbf {A}}^{gn} &{} {\mathbf {A}}^{gg} \end{array} \right]$$ is the inverse of the pedigree relationship matrix, ${\mathbf {A}}_{gg}$ is the pedigree relationship between genotyped animals, and ${\mathbf {G}}= \frac{1-w}{m} {\mathbf {Z}}\mathbf {Z'} + w {\mathbf {A}}_{gg}$ is the genomic relationship matrix with *w* being the proportion of variance (due to additive genetic effects) considered as residual polygenic effects, and $m=2\sum p_{o}\left( 1-p_{o}\right)$ with $p_{o}$ being the allele frequency of the $o{\text{th}}$ SNP. The matrix ${\mathbf {Z}}$ contains the SNP genotypes (coded as 0 for one homozygous genotype, 1 for the heterozygous genotype, or 2 for the alternate homozygous genotype) centered by their observed means.

The vector of solutions is equal to $${\mathbf {x}}_{G} = \left[ \begin{array}{c} \hat{\varvec{\upbeta }}\\ \hat{{\mathbf {u}}}_{n}\\ \hat{{\mathbf {u}}}_{g}\\ \end{array} \right]$$, where $\varvec{\upbeta }$ is the vector of fixed effects, ${\mathbf {u}}_{n}$ is the vector of additive genetic effects for non-genotyped animals, and ${\mathbf {u}}_{g}$ is the vector of additive genetic effects for genotyped animals.

#### ssSNPBLUP

For ssSNPBLUP [6], the vector ${\mathbf {b}}_{L}$ is equal to $${\mathbf {b}}_{L}= \left[ \begin{array}{c} {\mathbf {X}}^{'}{\mathbf {R}}^{-1}{\mathbf {y}} \\ {\mathbf {W}}^{'}_{n}{\mathbf {R}}^{-1}_{n}{\mathbf {y}}_{n} \\ {\mathbf {W}}^{'}_{g}{\mathbf {R}}^{-1}_{g}{\mathbf {y}}_{g} \\ {\mathbf {0}} \end{array} \right]$$.

The coefficient matrix ${\mathbf {C}}_{L}$ is equal to:

$$\begin{aligned} {\mathbf {C}}_{L}= \left[ \begin{array}{cccc} {\mathbf {X}}^{'}{\mathbf {R}}^{-1}{\mathbf {X}} &{} {\mathbf {X}}^{'}_{n}{\mathbf {R}}^{-1}_{n}{\mathbf {W}}_{n} &{} {\mathbf {X}}^{'}_{g}{\mathbf {R}}^{-1}_{g}{\mathbf {W}}_{g} &{} {\mathbf {0}} \\ {\mathbf {W}}^{'}_{n}{\mathbf {R}}^{-1}_{n}{\mathbf {X}}_{n} &{} {\mathbf {W}}^{'}_{n}{\mathbf {R}}^{-1}_{n}{\mathbf {W}}_{n}+{\mathbf {A}}^{nn}\sigma _{u}^{-2} &{} {\mathbf {A}}^{ng}\sigma _{u}^{-2} &{} {\mathbf {0}} \\ {\mathbf {W}}^{'}_{g}{\mathbf {R}}^{-1}_{g}{\mathbf {X}}_{g} &{} {\mathbf {A}}^{gn} \sigma _{u}^{-2} &{} {\mathbf {W}}^{'}_{g}{\mathbf {R}}^{-1}_{g}{\mathbf {W}}_{g}+ \left( {\mathbf {A}}^{gg} + {\varvec{\Sigma }}_{L,11} \right) \sigma _{u}^{-2} &{} {\varvec{\Sigma }}_{L,12} \sigma _{u}^{-2}\\ {\mathbf {0}} &{} {\mathbf {0}} &{} {\varvec{\Sigma }}_{L,21} \sigma _{u}^{-2}&{} {\varvec{\Sigma }}_{L,22} \sigma _{u}^{-2} \end{array} \right] \end{aligned}$$

where $${\varvec{\Sigma }}_{L}= \left[ \begin{array}{cccc} {\varvec{\Sigma }}_{L,11} &{}{\varvec{\Sigma }}_{L,12} \\ {\varvec{\Sigma }}_{L,21} &{}{\varvec{\Sigma }}_{L,22} \end{array} \right] = \left[ \begin{array}{cccc} \left( \frac{1}{w}-1 \right) {\mathbf {A}}^{-1}_{gg} &{} -\frac{1}{w}{\mathbf {A}}_{gg}^{-1}{\mathbf {Z}} \\ -\frac{1}{w}{\mathbf {Z}}^{'}{\mathbf {A}}_{gg}^{-1} &{} \frac{1}{w}{\mathbf {Z}}^{'}{\mathbf {A}}_{gg}^{-1}{\mathbf {Z}}+\frac{m}{1-w}{\mathbf {I}} \end{array} \right]$$.

The vector ${\mathbf {x}}_{L}$ is equal to $${\mathbf {x}}_{L}= \left[ \begin{array}{c} \hat{\varvec{\upbeta }}\\ \hat{{\mathbf {u}}}_{n}\\ \hat{{\mathbf {u}}}_{g}\\ \hat{{\mathbf {g}}} \end{array} \right]$$ where ${\mathbf {g}}$ is the vector of SNP effects.

The equivalence between ssGBLUP and ssSNPBLUP can be shown by absorbing the equations of the SNP effects of ssSNPBLUP and by using the Woodbury matrix identity [16]:

$$\begin{aligned} {\mathbf {G}}^{-1} - {\mathbf {A}}_{gg}^{-1}= & {} \left( \frac{1-w}{m} {\mathbf {Z}}\mathbf {Z'} + w {\mathbf {A}}_{gg} \right) ^{-1} - {\mathbf {A}}_{gg}^{-1} \\= & {} \left( \frac{1}{w} - 1 \right) {\mathbf {A}}_{gg}^{-1} - \frac{1}{w} {\mathbf {A}}_{gg}^{-1} {\mathbf {Z}} \left( \frac{1}{w} \mathbf {Z'} {\mathbf {A}}_{gg}^{-1} {\mathbf {Z}} + \frac{m}{1-w} {\mathbf {I}} \right) ^{-1} \mathbf {Z'} {\mathbf {A}}_{gg}^{-1}\frac{1}{w}. \end{aligned}$$

### PCG method

A PCG method is an iterative method that uses successive approximations to obtain more accurate solutions for a linear system at each iteration step [17]. In our implementation, the preconditioned system of linear equations of ssGBLUP and of ssSNPBLUP required by the PCG method has the form (with subscripts omitted):

1 $$\begin{aligned} \tilde{{\mathbf {M}}}^{-1}{\mathbf {C}}{\mathbf {x}}=\tilde{{\mathbf {M}}}^{-1}{\mathbf {b}}, \end{aligned}$$

where $\tilde{{\mathbf {M}}} = {\mathbf {D}} {\mathbf {M}}$ with ${\mathbf {M}}$ being a (block-)diagonal preconditioner defined below separately for each analysis, and ${\mathbf {D}}$ being an identity matrix for ssGBLUP, or a second-level diagonal preconditioner for ssSNPBLUP as proposed by Vandenplas et al. [10].

### Termination criteria

The relative error in ${\mathbf {x}}$ at the *i*-th iteration of the PCG method is defined as:

2 $$\begin{aligned} e_{r,i}=\frac{\Vert {\mathbf {x}}-\hat{{\mathbf {x}}}_{i} \Vert }{\Vert {\mathbf {x}}\Vert }, \end{aligned}$$

where $\hat{{\mathbf {x}}}_{i}$ is an approximate solution of ${\mathbf {x}}$ at the *i*-th iteration and $\Vert . \Vert$ is the 2-norm.

Unfortunately, because the true solution ${\mathbf {x}}$ is unknown, the relative error in ${\mathbf {x}}$ ($e_{r,i}$) cannot be computed and used as termination criterion. Therefore, alternative termination criteria must be used. A good termination criterion is important for iterative solvers, and should identify when $e_{r,i}$ is small enough to stop the iterative process. If this is not the case, the iterative process might be stopped too soon, resulting in useless approximate solutions, or take an unnecessary long time or never stop [18]. Relative termination criteria as those proposed below are scaling invariant, and are therefore usually preferred over absolute termination criteria.

In animal breeding, the PCG method is often stopped when the relative residual (denoted by CR) is lower than or equal to a pre-defined threshold $\epsilon _{CR}$, that is [11, 12]:

3 $$\begin{aligned} \frac{\Vert {\mathbf {r}}_{i} \Vert }{\Vert {\mathbf {b}}\Vert } \le \epsilon _{CR}, \end{aligned}$$

where $${\mathbf {r}}_{i} = {\mathbf {b}} - {\mathbf {C}} \hat{{\mathbf {x}}}_{i}$$.

It can be shown that the termination criterion CR is related to the relative error in ${\mathbf {x}}$, $e_{r,i}$ (Eq. 2), as follows [19] (see Additional file 1 for a derivation):

$$\begin{aligned} e_{r,i} \le \kappa \left( {\mathbf {C}} \right) \frac{\Vert {\mathbf {r}}_{i} \Vert }{\Vert {\mathbf {b}}\Vert }, \end{aligned}$$

where $\kappa \left( {\mathbf {C}} \right)$ is the effective spectral condition number of ${\mathbf {C}}$ defined as the ratio of its largest to smallest positive eigenvalues [20].

There are several drawbacks of the termination criterion CR (Eq. 3). First, the termination criterion CR may be difficult to satisfy when ${\mathbf {C}}$ is very ill-conditioned, and thus when $\kappa \left( {\mathbf {C}} \right)$ is large [18]. Second, the comparison of the convergence rates of the PCG method applied to different systems of equations may lead to wrong conclusions if $\kappa \left( {\mathbf {C}} \right)$ associated with the compared systems are very different. Indeed, using the same threshold $\epsilon _{CR}$ for the compared systems would result in PCG methods stopping at a same level of CR but at different levels of $e_{r}$. Finally, it is worth noting that $\kappa \left( {\mathbf {C}} \right)$ could be estimated at low costs during an unpreconditioned conjugate gradient (CG) process based on the equivalence of the CG and Lanczos methods [18, 21]. However, this study focuses on PCG methods for solving preconditioned systems of equations. In this context, the correction of the termination criterion CR by $\kappa \left( {\mathbf {C}} \right)$ cannot be applied because $\kappa \left( {\mathbf {C}} \right)$ cannot be computed easily for most large systems of equations.

Another termination criterion that is often used in animal breeding, is the relative difference between two consecutive iterates (denoted by CD) of the PCG method, that is [11]:

4 $$\begin{aligned} \frac{\Vert \hat{{\mathbf {x}}}_{i-1}-\hat{{\mathbf {x}}}_{i} \Vert }{\Vert \hat{{\mathbf {x}}}_{i} \Vert } \le \epsilon _{CD}. \end{aligned}$$

A drawback of the termination criterion CD is that it is not related to the relative error in ${\mathbf {x}}$, $e_{r,i}$. Therefore the termination criterion CD could be satisfied while $e_{r,i}$ is still large.

A third termination criterion (denoted by CK) is defined as follows [21, 22]:

5 $$\begin{aligned} \frac{1}{\mu _{1}} \frac{\Vert \tilde{{\mathbf {M}}}^{-1} {\mathbf {r}}_{i} \Vert }{\Vert \hat{{\mathbf {x}}}_{i} \Vert } \le \epsilon _{CK}, \end{aligned}$$

where $\mu _{1}$ is the smallest active eigenvalue (i.e. the smallest positive eigenvalue that influences the convergence [23]) of $\tilde{{\mathbf {M}}}^{-1}{\mathbf {C}}$.

It can be shown that the termination criterion CK is related to the relative error in ${\mathbf {x}}$, $e_{r,i}$ (Eq. 2), as follows [21, 22]:

$$\begin{aligned} e_{r,i} \le \frac{1}{\mu _{1}} \frac{\Vert \tilde{{\mathbf {M}}}^{-1} {\mathbf {r}}_{i} \Vert }{\Vert \hat{{\mathbf {x}}}_{i} \Vert }. \end{aligned}$$

Therefore, this termination criterion CK allows the user to specify the desired relative accuracy $\epsilon _{CK}$ in the computed solution $\hat{{\mathbf {x}}}_{i}$. To our knowledge, the termination criterion CK was never applied in animal breeding, and is scarcely used in other fields.

Finally, we introduce a fourth termination criterion (denoted by CM) defined as follows (see Additional file 2 for a derivation):

6 $$\begin{aligned} \kappa \left( \tilde{{\mathbf {M}}}^{-1}{\mathbf {C}} \right) \frac{\Vert \tilde{{\mathbf {M}}}^{-1}{\mathbf {r}}_{i} \Vert }{\Vert \tilde{{\mathbf {M}}}^{-1}{\mathbf {b}}\Vert } \le \epsilon _{CM}, \end{aligned}$$

where $\kappa \left( \tilde{{\mathbf {M}}}^{-1}{\mathbf {C}} \right)$ is the effective spectral condition number of $\tilde{{\mathbf {M}}}^{-1}{\mathbf {C}}$.

It can be shown that the termination criterion CM is related to the relative error in ${\mathbf {x}}$, $e_{r,i}$ (Eq. 2), as follows (see Additional file 2 for a derivation):

$$\begin{aligned} e_{r,i} \le \kappa \left( \tilde{{\mathbf {M}}}^{-1}{\mathbf {C}} \right) \frac{\Vert \tilde{{\mathbf {M}}}^{-1}{\mathbf {r}}_{i} \Vert }{\Vert \tilde{{\mathbf {M}}}^{-1}{\mathbf {b}}\Vert }. \end{aligned}$$

Like the termination criterion CK, the termination criterion CM is defined at the scale of the relative accuracy in ${\mathbf {x}}$. Thus, the termination criterion CM allows the user to specify the desired relative accuracy in ${\mathbf {x}}$. The termination criterion CM can be also considered as being the termination criterion CR applied to the preconditioned system of equations, instead of the system of equations directly (1). Finally, it is worth noting that the termination criterion CM is equal to the relative error in ${\mathbf {x}}$ if $\tilde{{\mathbf {M}}}={\mathbf {C}}$.

The termination criteria CK and CM require $\mu _{1}$ and $\kappa \left( \tilde{{\mathbf {M}}}^{-1}{\mathbf {C}} \right)$ for their computation. The estimates of $\mu _{1}$ and of $\kappa \left( \tilde{{\mathbf {M}}}^{-1}{\mathbf {C}} \right)$ can be obtained at low costs during the PCG process using the Lanczos method based on information obtained from the PCG method, e.g., as proposed by Kaasschieter [21] or as described below.

#### Relationships between termination criteria applied to ssGBLUP and ssSNPBLUP

When comparing the termination criterion CR between ssGBLUP and ssSNPBLUP, it is worth noting that $\Vert {\mathbf {b}}_{G} \Vert = \Vert {\mathbf {b}}_{L} \Vert$ because all entries of the right-hand sides are the same, except for the entries corresponding to the SNP equations that are equal to 0. Therefore, when estimates for the common entries between $\hat{{\mathbf {x}}}_{G}$ and $\hat{{\mathbf {x}}}_{L}$ are equal (i.e., when $\hat{\varvec{\upbeta }}_{G}=\hat{\varvec{\upbeta }}_{L}$, $\hat{{\mathbf {u}}}_{n,G}=\hat{{\mathbf {u}}}_{n,L}$, and $\hat{{\mathbf {u}}}_{g,G}=\hat{{\mathbf {u}}}_{g,L}$), any observed differences between CR for ssGBLUP and for ssSNPBLUP are a consequence of the presence of the estimates of SNP effects in the solution vector of ssSNPBLUP $\hat{{\mathbf {x}}}_{L}$. More specifically, it can be shown in this case that the differences between CR for ssGBLUP and for ssSNPBLUP can be explained by the error due to the PCG iterative process in estimating the SNP effects $\hat{{\mathbf {g}}}$, $\epsilon _{L}$:

$$\epsilon _{L} = \hat{{\mathbf {g}}}_{L} - \left( \frac{1}{w}{\mathbf {Z}}^{'}{\mathbf {A}}_{gg}^{-1}{\mathbf {Z}}+\frac{m}{1-w}{\mathbf {I}}\right) ^{-1} \frac{1}{w}{\mathbf {A}}_{gg}^{-1}{\mathbf {Z}} \hat{{\mathbf {u}}}_{g,L}$$.

Indeed, after some derivations (see Additional file 3 for details), we can show that:

7 $$\begin{aligned} \Vert {\mathbf {r}}_{L} \Vert= & {} \Vert \left[ \begin{array}{c} {\mathbf {r}}_{G} \\ {\mathbf {0}} \end{array} \right] + \left[ \begin{array}{c} \left[ \begin{array}{c} {\mathbf {0}}\\ \frac{1}{w}{\mathbf {A}}_{gg}^{-1}{\mathbf {Z}} \sigma _{u}^{-2} \epsilon _{L} \end{array} \right] \\ \left( \frac{1}{w}{\mathbf {Z}}^{'}{\mathbf {A}}_{gg}^{-1}{\mathbf {Z}}+\frac{m}{1-w}{\mathbf {I}}\right) \sigma _{u}^{-2} \epsilon _{L} \end{array} \right] \Vert , \end{aligned}$$

where ${\mathbf {r}}_{L} = {\mathbf {b}}_{L} - {\mathbf {C}}_{L} \hat{{\mathbf {x}}}_{L}$ is the residual of the ssSNPBLUP system of equations, and ${\mathbf {r}}_{G} = {\mathbf {b}}_{G} - {\mathbf {C}}_{G} \hat{{\mathbf {x}}}_{G}$ is the residual of the ssGBLUP system of equations.

Finally, $\Vert {\mathbf {x}}_{G} \Vert$ is approximately equal to $\Vert {\mathbf {x}}_{L} \Vert$ (similarly for $\Vert \hat{{\mathbf {x}}}_{G} \Vert$ and $\Vert \hat{{\mathbf {x}}}_{L} \Vert$) when the common entries of both vectors are equal. Indeed, the only different entries between these two vectors are the additional estimates of SNP effects that are of several thousand orders of magnitude lower than solutions of other fixed and random effects (e.g., genetic additive effects). Furthermore, the number of SNP effects (e.g., 50,000) is relatively low in comparison to the total number of equations in evaluations with a deep pedigree (e.g., with millions of animals). Therefore, the termination criterion $e_{r}$ for ssGBLUP and for ssSNPBLUP should have similar values, provided that the common solutions of both systems are similar.

### Data

The first data set used in this study, hereafter referred to as the FIN data set, was provided by Topigs Norsvin (The Netherlands). The other two data sets used in this study, hereafter referred to as KAR and LON data sets, were provided by CRV BV (The Netherlands).

The FIN data set and associated variance components were extracted from an 11-trait genetic evaluation for grower-finisher traits. After extraction, the data file included 752,067 records with a single record per animal (all born before July 2017), and all records had missing observations for at least one trait. The pedigree included 835,562 animals. The genotypes included 23,102 segregating SNPs, and were associated with 38,488 animals. The 11-trait mixed model included random effects (litter, pen, group, additive genetic and residual), fixed co-variables [weight at start, at halfway, and at the end of the feed intake trajectory, weight (linear and quadratic) nested within farm-line-sex, probability of the sire passing the favorable allele for insulin-growth factor 2 (IGF2)] and fixed cross-classified effects (trial, farm-line-sex, compartment within farm). The traits included average daily gain measured across two time periods, back fat, loin depth, and feed intake across the entire testing period (all as separate traits for purebred and crossbred animals), and feed intake across the second half of the testing period, for purebred animals only.

The KAR data set and associated variance components were from the four-trait routine genetic evaluation of December 2019 for temperament and milking speed of dairy cattle for the Netherlands and the Flemish region of Belgium [24, 25]. Performances in each of these two countries were considered as different traits. The data file included 4,058,154 records with a single record per animal. The pedigree included 6,344,482 animals. The genotypes included 37,995 segregating SNPs, and were associated with 123,644 animals. The four-trait mixed model included random effects (additive genetic and residual), fixed co-variables (heterosis and recombination) and fixed cross-classified effects (herd × year × season at classification, age at classification, lactation stage at classification, milk yield and month of calving) [24, 25].

The LON data set and associated variance components were from the univariate routine genetic evaluation of August 2019 for longevity of dairy cattle for the Netherlands and the Flemish region of Belgium [26, 27]. The data file included 408,107,042 records associated with 12,528,520 animals. The pedigree included 14,589,796 animals. The genotypes included 37,995 segregating SNPs, and were associated with 120,000 animals that were randomly selected among 192,714 genotyped animals. The univariate random regression mixed model included random effects (Legendre polynomials (5th order) and residual), fixed co-variables (heterosis and recombination) and fixed cross-classified effects (herd × year × season × lactation stage, year × season × age of first calving × within-herd production level × lactation-stage, herd size change) [26, 27].

### Analyses

The ssGBLUP and ssSNPBLUP systems for the three data sets were solved using a Fortran 2003 program that is described in Vandenplas et al. [28]. All real vectors and matrices (including the preconditioner) were stored in-memory using double-precision real arrays.

For the FIN data set, the preconditioner ${\mathbf {M}}$ had a block-diagonal structure that included all equations for the fixed effects, and a block-diagonal structure for the random effects with blocks corresponding to equations for all traits within a level (e.g., an animal). For the KAR data set, the preconditioner ${\mathbf {M}}$ included only the diagonal elements of the coefficient matrix for the fixed effects, and a block-diagonal structure for the random effects with blocks corresponding to equations for different traits within a level (see [9] for more details). For the LON data set, two different preconditioners were tested to evaluate their impact on convergence. In the first case, the preconditioner ${\mathbf {M}}$ had a diagonal structure for all effects. Hereafter we refer to this evaluation as the LON evaluation. In the second case, the preconditioner ${\mathbf {M}}$ had a diagonal structure for the fixed effects, and blocks of elements for the random effects with blocks corresponding to equations for the different Legendre polynomials within a level (i.e. an animal). Hereafter we refer to this evaluation as the LON + block evaluation. For the three data sets, the proportion of variance (due to additive genetic effects) considered as residual polygenic effects, *w*, was assumed to be equal to 0.10 or 0.30 to evaluate its impact on convergence.

For analyzing the different termination criteria, all systems of equations were solved twice with the PCG method. All PCG processes iterated until the termination criterion CM or CK was $\le 5.*10^{-3}$, ensuring a relative error in the solution $\le 5.*10^{-3}$. Each solution vector obtained from the first iterative process, hereafter called manufactured solution, was pre-multiplied by the corresponding coefficient matrix to obtain a manufactured right-hand side. The systems of equations were then solved a second time after replacing the right-hand sides computed from the datasets by the manufactured right-hand sides. The manufactured solutions were therefore the true solutions of these systems of equations with manufactured right-hand sides. All results presented in this study are related to these systems of equations.

The smallest and largest eigenvalues that influence the convergence were approximated by the smallest and largest Ritz values, respectively. These extremal Ritz values were obtained every 200-th iteration, starting at iteration 200, using the Lanczos method based on information obtained from the PCG method [21]. In our approach, the main computational cost consisted of the eigendecomposition of a tridiagonal matrix of size equal to the number of iterations and computed following Eq. 2.3 in Kaasschieter [21].

The smallest Ritz value was used to estimate $\mu _{1}$, needed for the termination criterion CK, and the ratio of the largest and smallest Ritz values was used to approximate $\kappa \left( \tilde{{\mathbf {M}}}^{-1}{\mathbf {C}} \right)$, needed for the termination criterion CM. Our initial analyses showed that the termination criteria CK and CM could result in stopping the iterative process too soon (i.e. before a desired level of accuracy of the solutions is achieved), because the smallest Ritz values computed at the beginning of the iterative process are poor estimates of the smallest active eigenvalues of the preconditioned coefficient matrix. Therefore, starting values for $\mu _{1}$ and $\kappa \left( \tilde{{\mathbf {M}}}^{-1}{\mathbf {C}} \right)$ were provided to the PCG method. These starting values for $\mu _{1}$ ($\kappa \left( \tilde{{\mathbf {M}}}^{-1}{\mathbf {C}} \right)$) were replaced by the Ritz value-based estimates when they became larger (smaller) than their corresponding estimates. Based on previous experiences, the starting values used for $\mu _{1}$ ($\kappa \left( \tilde{{\mathbf {M}}}^{-1}{\mathbf {C}} \right)$) were set to $10^{-6}$ ($10^{6}$) for FIN, $10^{-5}$ ($1.5*10^{6}$) for KAR, $10^{-5}$ ($10^{6}$) for LON, and $2*10^{-5}$ ($10^{5}$) for LON + block.

The solution vector was saved in a binary file every 100-th iteration starting at iteration 200. After termination, using the manufactured solutions as true solutions, the relative error in ${\mathbf {x}}$, $e_{r,i}$, was computed every 100-th iteration. The termination criteria CR and CD, as well as $\frac{\Vert \tilde{{\mathbf {M}}}^{-1}{\mathbf {r}}_{i} \Vert }{\Vert \hat{{\mathbf {x}}}_{i} \Vert }$ and $\frac{\Vert \tilde{{\mathbf {M}}}^{-1}{\mathbf {r}}_{i} \Vert }{\Vert \tilde{{\mathbf {M}}}^{-1}{\mathbf {b}}\Vert }$, were computed at each iteration. After the last PCG iteration was finished, the largest and smallest Ritz values were computed. The termination criteria CK and CM were then retrospectively computed for all iterations, with $\mu _{1}$ being approximated by the smallest Ritz value, and $\kappa \left( \tilde{{\mathbf {M}}}^{-1}{\mathbf {C}} \right)$ by the ratio of the largest and smallest Ritz values. The termination criterion CR for ssSNPBLUP was also computed by excluding equations for SNP effects.

Finally, from a practical point of view, a relative error in the solution $\le 5.*10^{-3}$ might not be required in routine evaluations, and the iterative process could be stopped sooner. We investigated this assumption by determining the number of iterations needed to achieve a maximal absolute difference between intermediate and true genetic effects (or Legendre polynomials) lower than 1% of a genetic standard deviation for all the traits. The number of iterations needed to achieve a Pearson correlation greater than 0.99990 between intermediate and true genetic effects (or Legendre polynomials) for each trait separately was also determined.

## Results

### Ritz values and effective spectral condition numbers

**Fig. 1:**
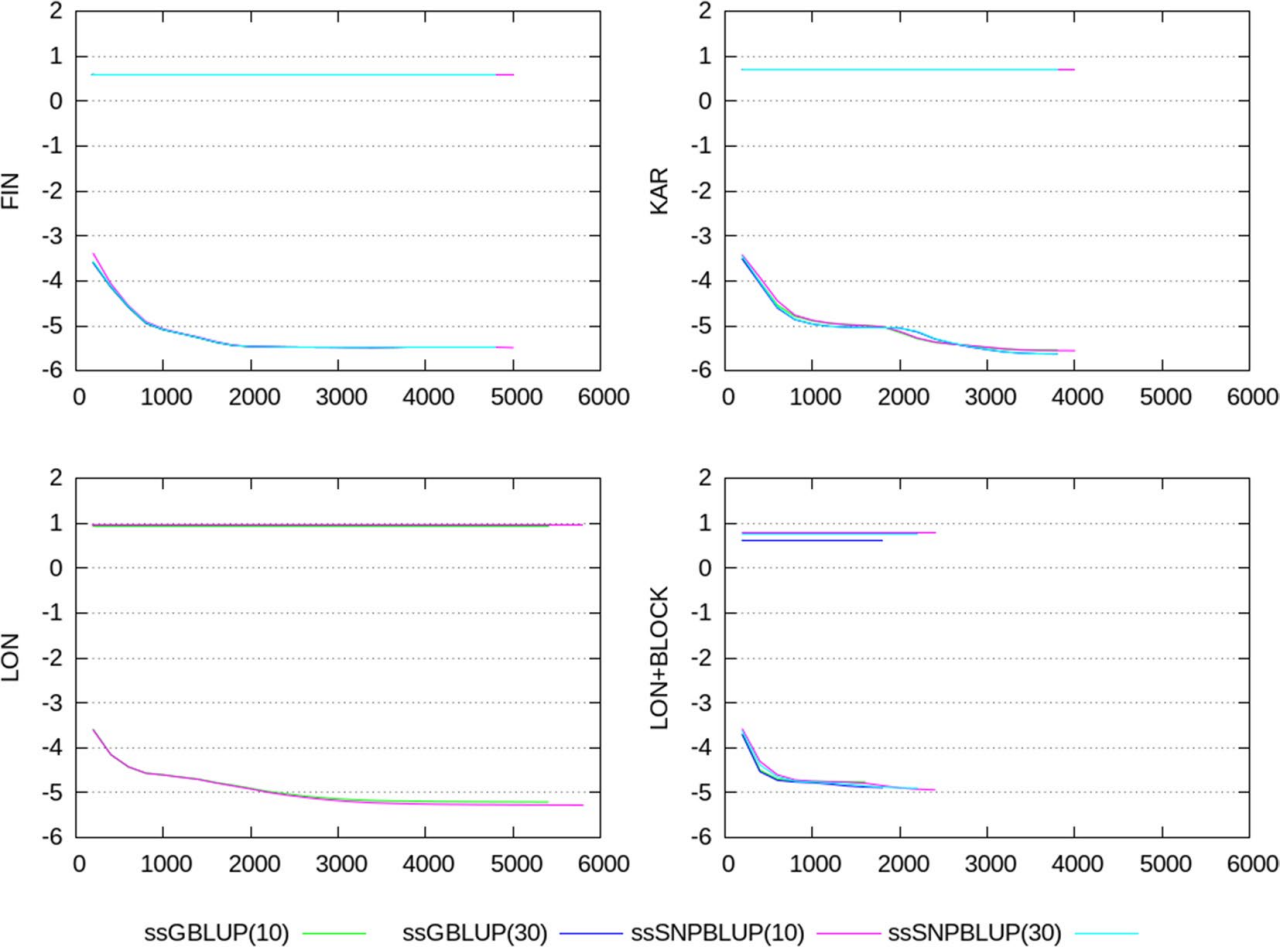
Logarithm of the smallest and largest Ritz values (on the y-axis) for the different evaluations. Smallest Ritz values are depicted for ssGBLUP with a proportion of residual polygenic variance equal to 10%, for ssGBLUP with a proportion of residual polygenic variance equal to 30%, for ssSNPBLUP with a proportion of residual polygenic variance equal to 10%, and ssSNPBLUP with a proportion of residual polygenic variance equal to 30%

The Ritz values computed at different iterations are depicted in Fig. 1. For the four evaluations, the largest Ritz values varied between 3.9 and 8.9, and are well estimated within less than 200 iterations (Fig. 1; Table 1). Furthermore, within each of the four evaluations, the largest Ritz values of equivalent ssGBLUP and ssSNPBLUP were almost equal. Regarding the smallest Ritz values, their estimates follow a similar pattern for ssGBLUP and ssSNPBLUP. Therefore, they are of similar order for each of the four evaluations, and varied between $10^{-5}$ and $10^{-6}$ after termination (Fig. 1; Table 1). In all cases, both the largest and the smallest Ritz values for ssGBLUP and ssSNPBLUP do not seem to be influenced by the proportion of variance assigned to the residual polygenic effects. Finally, similar extremal Ritz values associated with different ssGBLUP and ssSNPBLUP resulted similar estimates of effective spectral condition numbers for these linear systems (i.e. all around $10^{6}$; Table 1).

**Table 1:** Characteristics of systems for ssGBLUP and ssSNPBLUP

Evaluation	Model$^{\text{a}}$	#Equations	#Iterations	Smallest eig.	Largest eig.	${\kappa }$ $^{\text{b}}$
FIN	ssGBLUP (10)	11,373,208	4912	$3.270*10^{-6}$	3.924	$1.200*10^{6}$
	ssGBLUP (30)	11,373,208	4929	$3.269*10^{-6}$	3.934	$1.203*10^{6}$
	ssSNPBLUP (10)	11,627,330	5001	$3.270*10^{-6}$	3.921	$1.199*10^{6}$
	ssSNPBLUP (30)	11,627,330	4937	$3.269*10^{-6}$	3.931	$1.202*10^{6}$
KAR	ssGBLUP (10)	26,709,604	3902	$2.882*10^{-6}$	5.062	$1.757*10^{6}$
	ssGBLUP (30)	26,709,604	3968	$2.379*10^{-6}$	5.063	$2.128*10^{6}$
	ssSNPBLUP (10)	26,861,584	4037	$2.811*10^{-6}$	5.062	$1.801*10^{6}$
	ssSNPBLUP (30)	26,861,584	3988	$2.366*10^{-6}$	5.063	$2.140*10^{6}$
LVD	ssGBLUP (10)	96,688,714	5431	$6.248*10^{-6}$	8.635	$1.382*10^{6}$
	ssSNPBLUP (10)	96,916,684	5888	$5.312*10^{-6}$	8.935	$1.682*10^{6}$
LVD + block	ssGBLUP (10)	96,688,714	1761	$1.672*10^{-5}$	4.160	$2.488*10^{5}$
	ssGBLUP (30)	96,688,714	1959	$1.295*10^{-5}$	4.161	$3.212*10^{5}$
	ssSNPBLUP (10)	96,916,684	2542	$1.140*10^{-5}$	6.201	$5.441*10^{5}$
	ssSNPBLUP (30)	96,916,684	2336	$1.260*10^{-5}$	5.686	$4.513*10^{5}$

^a^Percentage of variance (due to additive genetic effects) explained by residual polygenic effects

^b^$\kappa$ = Effective spectral condition number of the preconditioned coefficient matrix

### Termination criteria

**Fig. 2:**
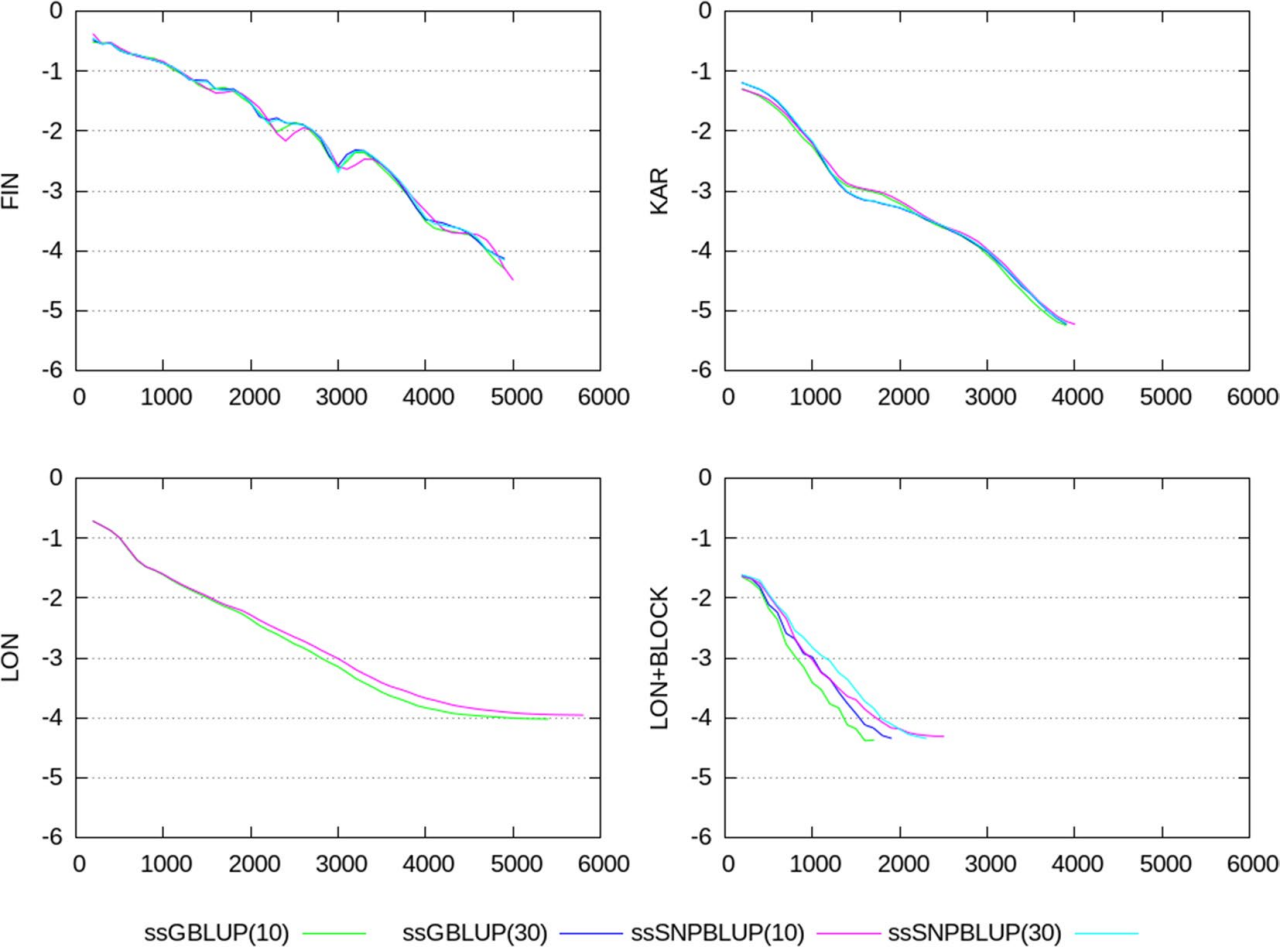
Relative errors in the solutions for the different evaluations. Relative errors in the solutions are depicted for ssGBLUP with a proportion of residual polygenic variance equal to 10%, for ssGBLUP with a proportion of residual polygenic variance equal to 30%, for ssSNPBLUP with a proportion of residual polygenic variance equal to 10%, and ssSNPBLUP with a proportion of residual polygenic variance equal to 30%

**Fig. 3:**
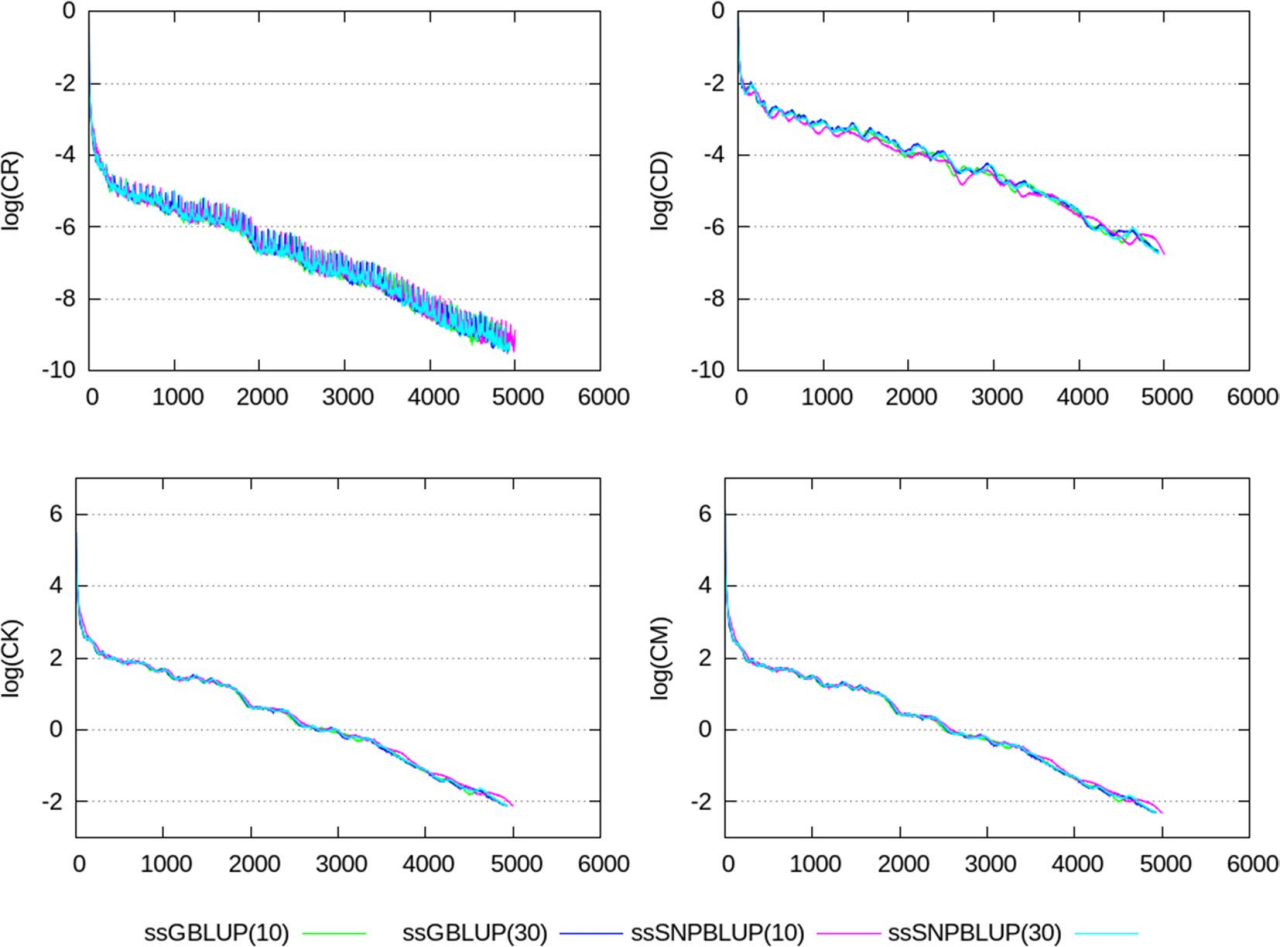
Termination criteria for the FIN data set. Termination criteria are depicted for ssGBLUP with a proportion of residual polygenic variance equal to 10%, for ssGBLUP with a proportion of residual polygenic variance equal to 30%, for ssSNPBLUP with a proportion of residual polygenic variance equal to 10%, and ssSNPBLUP with a proportion of residual polygenic variance equal to 30%

**Fig. 4:**
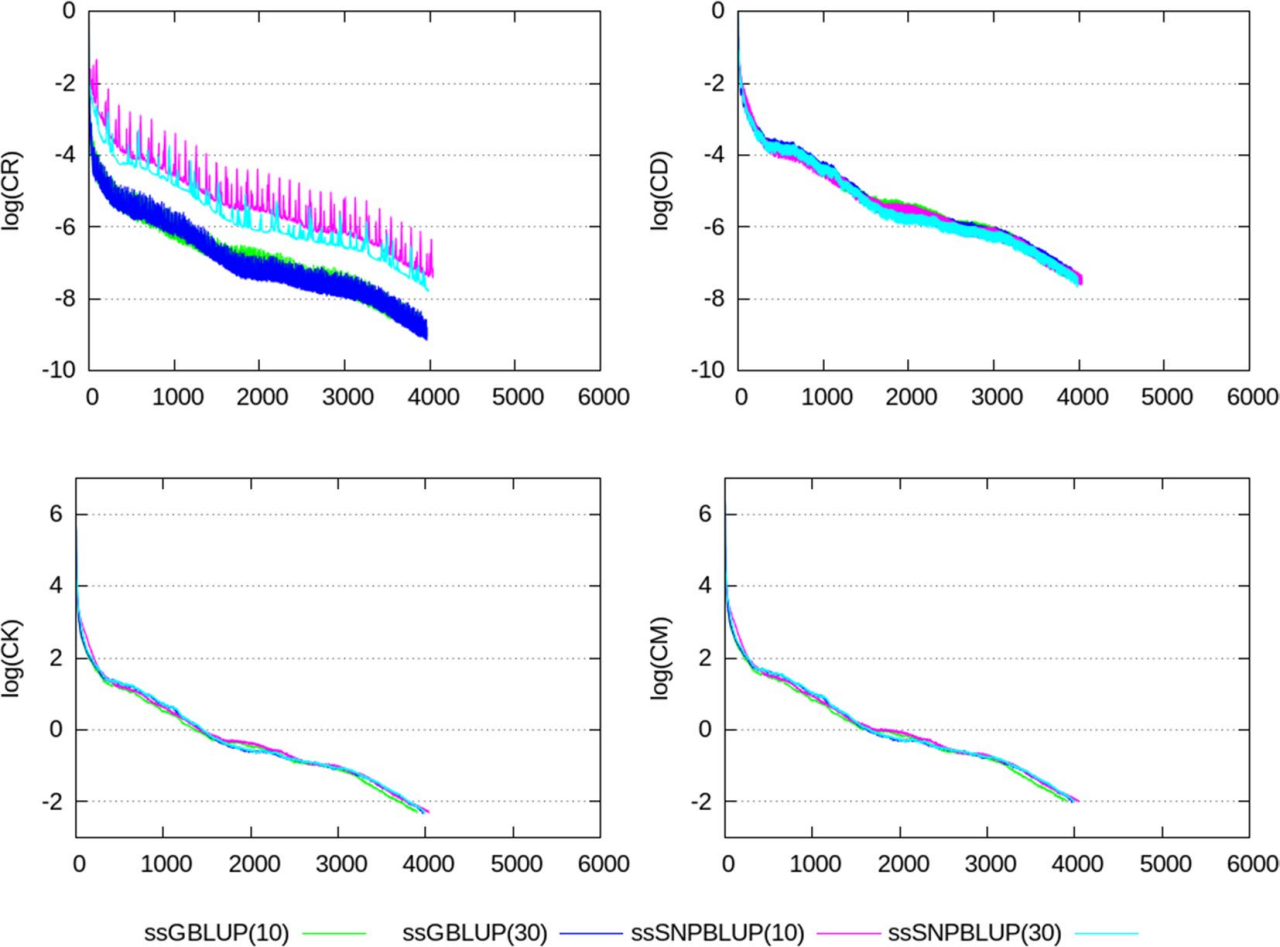
Termination criteria for the KAR data set. Termination criteria are depicted for ssGBLUP with a proportion of residual polygenic variance equal to 10%, for ssGBLUP with a proportion of residual polygenic variance equal to 30%, for ssSNPBLUP with a proportion of residual polygenic variance equal to 10%, and ssSNPBLUP with a proportion of residual polygenic variance equal to 30%

**Fig. 5:**
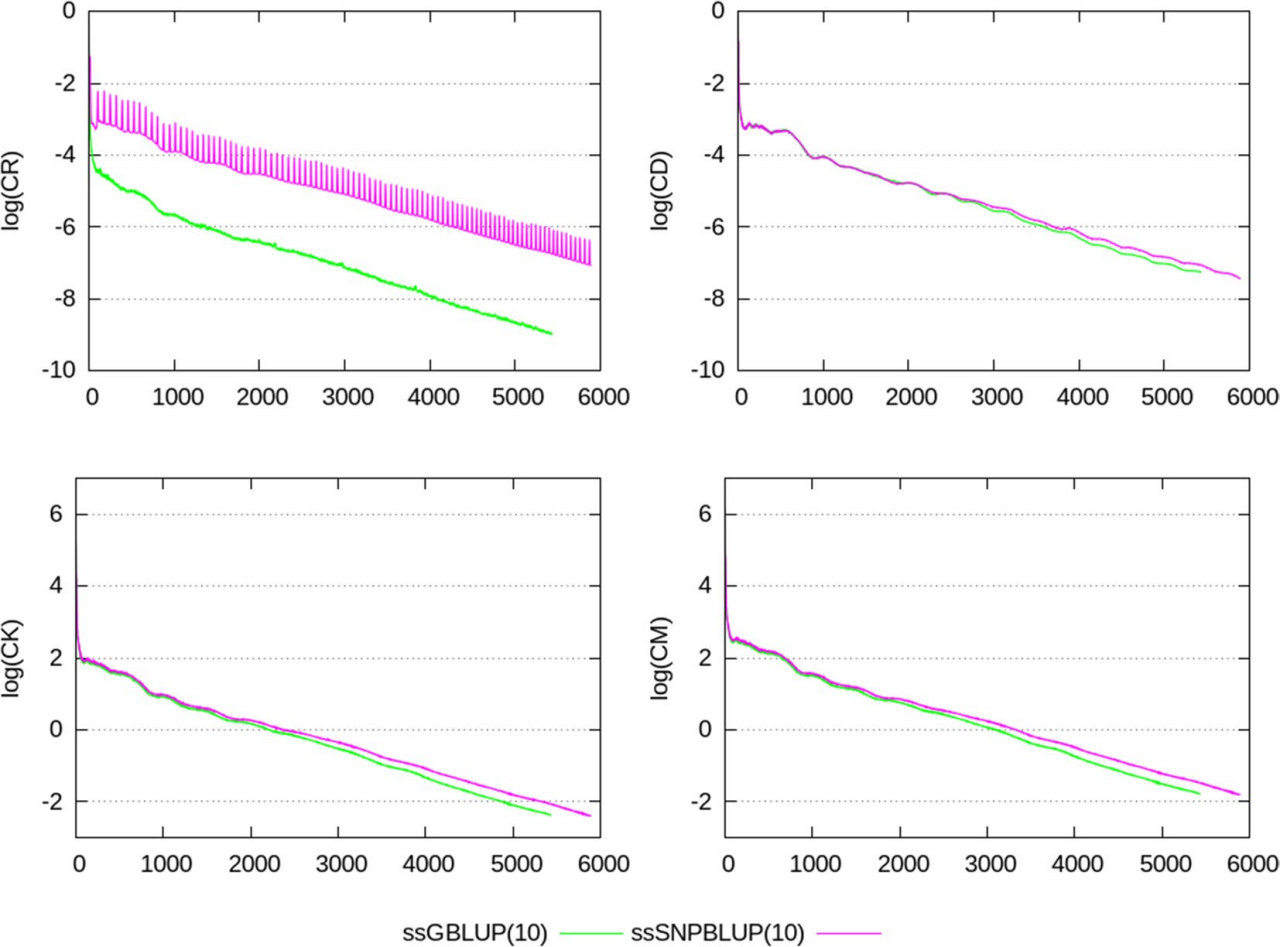
Termination criteria for the LON data set when using a diagonal preconditioner. Termination criteria are depicted for ssGBLUP with a proportion of residual polygenic variance equal to 10%, and ssSNPBLUP with a proportion of residual polygenic variance equal to 10%

**Fig. 6:**
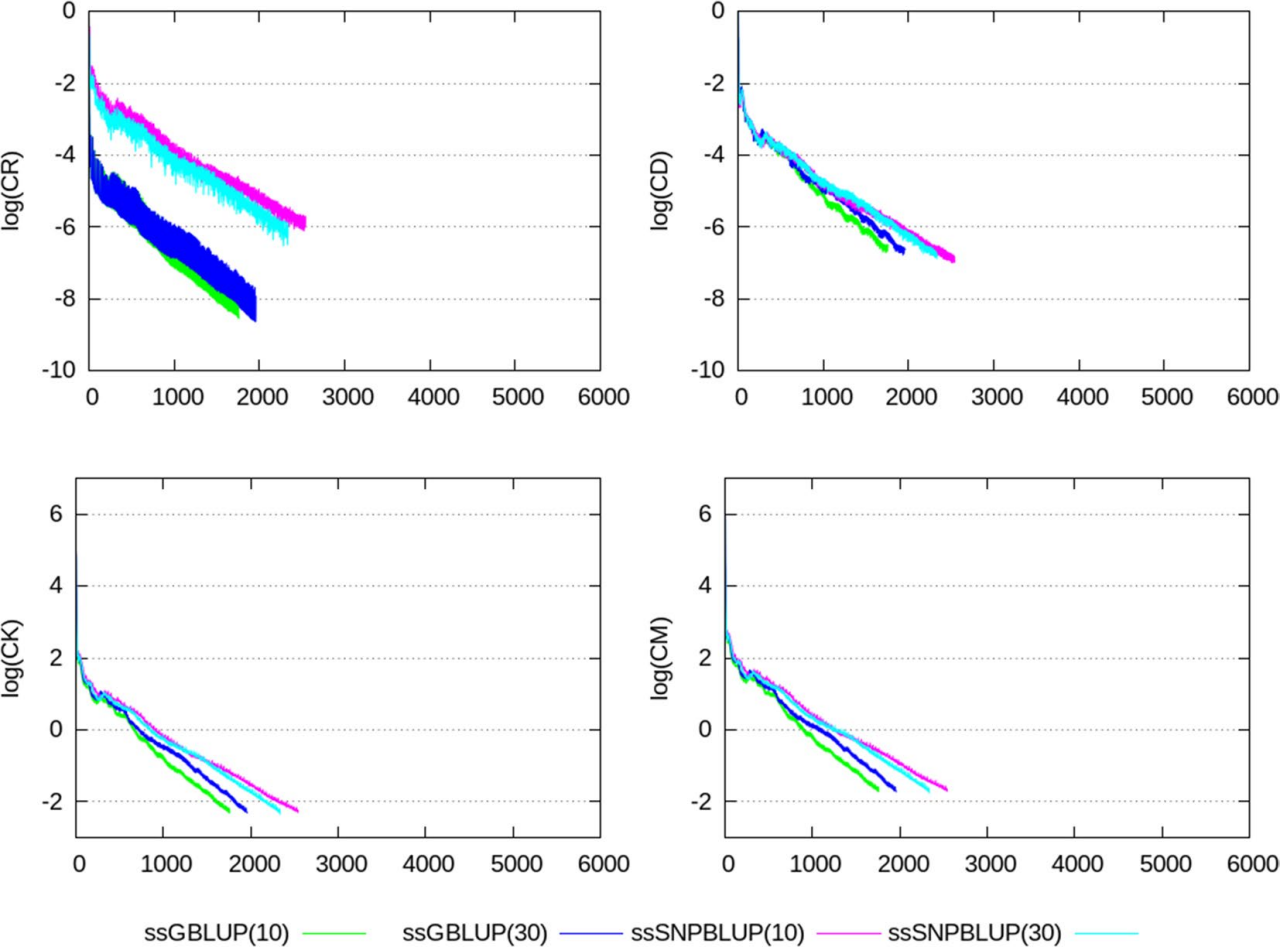
Termination criteria for the LON data set when using a block-diagonal preconditioner. Termination criteria are depicted for ssGBLUP with a proportion of residual polygenic variance equal to 10%, for ssGBLUP with a proportion of residual polygenic variance equal to 30%, for ssSNPBLUP with a proportion of residual polygenic variance equal to 10%, and ssSNPBLUP with a proportion of residual polygenic variance equal to 30%

**Fig. 7:**
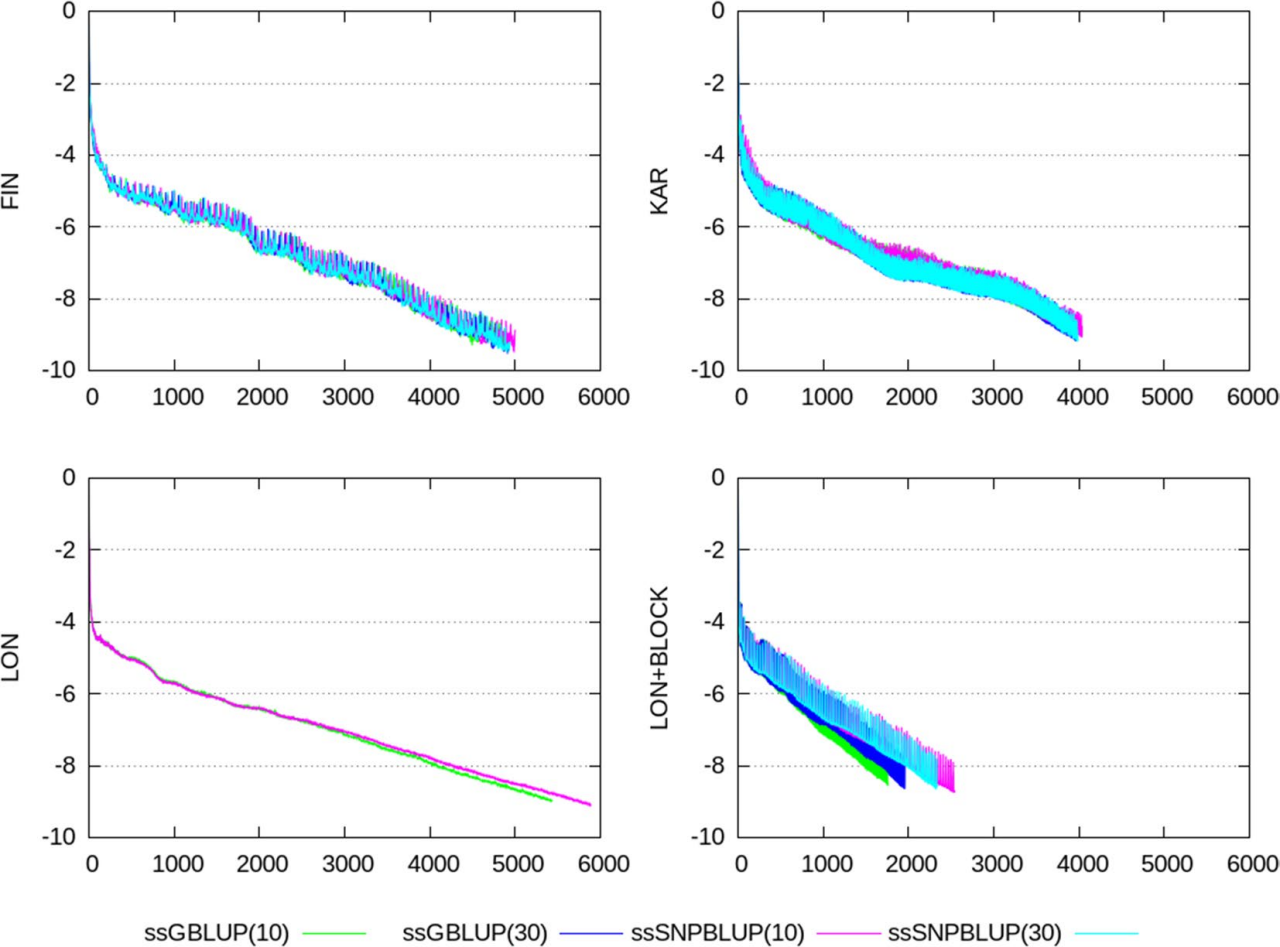
Logarithm of termination criterion CR computed by excluding the SNP effects for different evaluations. Termination criterion CR is depicted for ssGBLUP with a proportion of residual polygenic variance equal to 10%, for ssGBLUP with a proportion of residual polygenic variance equal to 30%, for ssSNPBLUP with a proportion of residual polygenic variance equal to 10%, and ssSNPBLUP with a proportion of residual polygenic variance equal to 30%

Termination criteria $e_{r}$, CD, CK, and CM, shown in Figs. 2, 3, 4, 5, 6, show similar patterns for ssGBLUP and ssSNPBLUP for the four evaluations, and independently of the proportion of additive genetic variance assigned to the residual polygenic effects. This is not the case for the termination criterion CR that is associated with a pattern for ssSNPBLUP applied to KAR, LON, and LON + block a few folds higher than the corresponding pattern of CR for ssGBLUP. This behavior is not observed for FIN. Excluding the equations of the SNP effects for computing the termination criterion CR for ssSNPBLUP resulted in a pattern similar to the corresponding pattern for CR of ssGBLUP for all four evaluations (Fig. 7).

Maximal absolute differences between intermediate and true genetic effects (or Legendre polynomials) lower than 1% of a genetic standard deviation for all traits were obtained for FIN after about 65% of the total number of iterations needed to satisfy a threshold of $5*10^{-3}$ for CK or CM, after around 35–47% for KAR, and after around 90% for LON and LON + block (Table 2). Even fewer iterations were needed to reach correlations between intermediate and true genetic effects (or Legendre polynomials) higher than 0.99990: around 53% of the total number of iterations for FIN, around 27% for KAR, and between 63 and 77% for LON and LON + block (Table 3).

**Table 2:** Number of iterations needed to reach a difference (for each trait) between intermediate and true estimates of genetic effects (or Legendre polynomials) lower than 1%

Evaluation	Model$^{\text{a}}$	# Iterations$^{\text{b}}$	$e_{r}$ $^{\text{c}}$	CR	CD	CK	CM
FIN	ssGBLUP (10)	3200	$4.432*10^{-3}$	$4.295*10^{-8}$	$1.134*10^{-5}$	0.105	0.327
	ssGBLUP (30)	3200	$4.864*10^{-3}$	$3.162*10^{-8}$	$1.922*10^{-5}$	0.181	0.444
	ssSNPBLUP (10)	3200	$2.724*10^{-3}$	$3.719*10^{-8}$	$1.871*10^{-5}$	0.155	0.448
	ssSNPBLUP (30)	3200	$4.600*10^{-3}$	$5.168*10^{-8}$	$1.536*10^{-5}$	0.172	0.412
KAR	ssGBLUP (10)	1800	$8.745*10^{-4}$	$6.520*10^{-8}$	$5.535*10^{-6}$	0.431	0.877
	ssGBLUP (30)	1400	$9.561*10^{-4}$	$7.483*10^{-7}$	$8.794*10^{-6}$	1.163	2.372
	ssSNPBLUP (10)	1900	$8.180*10^{-4}$	$6.845*10^{-6}$	$4.194*10^{-6}$	0.444	0.905
	ssSNPBLUP (30)	1500	$8.181*10^{-4}$	$4.504*10^{-6}$	$6.830*10^{-6}$	0.802	1.636
LON	ssGBLUP (10)	5200	$9.709*10^{-5}$	$1.590*10^{-9}$	$6.699*10^{-8}$	0.006	0.023
	ssSNPBLUP (10)	5600	$1.126*10^{-4}$	$6.966*10^{-7}$	$5.583*10^{-8}$	0.006	0.025
LON + block	ssGBLUP (10)	1600	$4.178*10^{-5}$	$1.868*10^{-8}$	$5.976*10^{-7}$	0.010	0.039
	ssGBLUP (30)	1800	$5.085*10^{-5}$	$5.054*10^{-9}$	$5.041*10^{-7}$	0.011	0.042
	ssSNPBLUP (10)	2000	$6.559*10^{-5}$	$4.742*10^{-6}$	$7.822*10^{-7}$	0.021	0.083
	ssSNPBLUP (30)	2100	$5.314*10^{-5}$	$1.372*10^{-6}$	$4.656*10^{-7}$	0.013	0.052

Values of termination criteria corresponding to the number of iterations are reported

$^{\text{a}}$Percentage of variance (due to additive genetic effects) explained by residual polygenic effects

$^{\text{b}}$The solutions were stored and evaluated every 100-th iteration

$^{\text{c}}$ $e_{r}$ = relative errors in the solutions

**Table 3:** Number of iterations needed to reach a Pearson correlation (for each trait) between intermediate and true estimates of genetic effects (or Legendre polynomials) greater than 0.99990

Evaluation	Model$^{\text{a}}$	# Iterations$^{\text{b}}$	$e_{r}$ $^{\text{c}}$	CR	CD	CK	CM
FIN	ssGBLUP (10)	2700	$9.547*10^{-3}$	$1.623*10^{-7}$	$4.237*10^{-5}$	0.241	0.749
	ssGBLUP (30)	2600	$1.282*10^{-2}$	$7.813*10^{-8}$	$3.195*10^{-5}$	0.327	0.802
	ssSNPBLUP (10)	2500	$9.372*10^{-3}$	$1.274*10^{-7}$	$5.587*10^{-5}$	0.530	1.533
	ssSNPBLUP (30)	2700	$1.069*10^{-2}$	$2.117*10^{-7}$	$4.144*10^{-5}$	0.335	0.805
KAR	ssGBLUP (10)	1100	$3.460*10^{-3}$	$3.293*10^{-7}$	$3.967*10^{-5}$	2.595	5.284
	ssGBLUP (30)	1100	$3.675*10^{-3}$	$1.063*10^{-6}$	$2.904*10^{-5}$	4.096	8.354
	ssSNPBLUP (10)	1100	$4.115*10^{-3}$	$1.851*10^{-5}$	$1.683*10^{-5}$	2.737	5.572
	ssSNPBLUP (30)	1100	$4.039*10^{-3}$	$1.135*10^{-5}$	$2.435*10^{-5}$	4.189	8.544
LON	ssGBLUP (10)	3900	$1.607*10^{-4}$	$1.540*10^{-8}$	$6.789*10^{-7}$	0.060	0.235
	ssSNPBLUP (10)	4100	$1.974*10^{-4}$	$1.849*10^{-6}$	$5.344*10^{-7}$	0.069	0.267
LON + block	ssGBLUP (10)	1300	$1.476*10^{-4}$	$2.273*10^{-8}$	$2.121*10^{-6}$	0.040	0.154
	ssGBLUP (30)	1500	$1.177*10^{-4}$	$1.987*10^{-8}$	$2.003*10^{-6}$	0.043	0.165
	ssSNPBLUP (10)	1600	$1.393*10^{-4}$	$1.549*10^{-5}$	$2.839*10^{-6}$	0.073	0.282
	ssSNPBLUP (30)	1800	$9.499*10^{-5}$	$5.961*10^{-6}$	$1.352*10^{-6}$	0.039	0.153

Values of termination criteria corresponding to the number of iterations are reported

$^{\text{a}}$Percentage of variance (due to additive genetic effects) explained by residual polygenic effects

$^{\text{b}}$The solutions were stored and evaluated every 100-th iteration

$^{\text{c}}$ $e_{r}$ = relative errors in the solutions

## Discussion

### Convergence behavior of the PCG method

The rate of convergence of CG-based methods depends not only on the effective spectral condition number of the (preconditioned) coefficient matrix of the system being solved, but also on the distribution of eigenvalues of the (preconditioned) coefficient matrix [23, 29]. For the data sets used in this study, the PCG methods applied to ssGBLUP and ssSNPBLUP show an approximately linear convergence behavior in the logarithm of the relative error in the solution ${\mathbf {x}}$, $e_{r}$. Similar convergence behaviors can be also observed for other termination criteria in this study, as well as in other studies that used the PCG method to solve pedigree BLUP or ssGBLUP (e.g., [11, 12, 30]). These approximately linear convergence behaviors suggest that the spectra of the preconditioned coefficient matrices are composed of eigenvalues that are well distributed across the whole spectrum. This implies that these spectra have no, or only a few, isolated eigenvalues [23]. Such a well-distributed spectrum and linear convergence were also observed by Vandenplas et al. [9] who computed the entire spectrum of a preconditioned coefficient matrix for a small system of ssSNPBLUP developed by Mantysaari and Stranden [31]. Therefore, the rate of convergence of the PCG method applied to ssGBLUP or ssSNPBLUP mainly depends on the effective spectral condition number of the associated preconditioned coefficient matrix, and not on its associated distribution of eigenvalues. It follows that the PCG method applied to different evaluations (ssGBLUP or ssSNPBLUP) associated with similar effective spectral condition numbers should result in similar rates of convergence, convergence behavior, and number of iterations to meet a defined threshold of a specific termination criterion, provided that the termination criterion does not depend on the properties of the different systems, or considers them adequately. This was the case for the evaluations FIN, KAR, and LON. The different ssGBLUP and ssSNPBLUP evaluations for LON + block were associated to slightly different effective spectral condition numbers, resulting in different numbers of iterations to satisfy the same termination criterion.

### Termination criteria

Four termination criteria were compared across different combinations of data sets and models. One of them, the termination criterion CR, is related to the residual of the system, and shows different patterns when applied to ssGBLUP and ssSNPBLUP. These different behaviors can be explained by the errors in the estimates of the SNP effects. Indeed, excluding the SNP equations from the termination criterion CR for ssSNPBLUP resulted in patterns similar to those of ssGBLUP (Eq. (7); Fig. 7). Using the termination criterion CR for ssSNPBLUP may lead to wrong conclusions, such as apparently non-accurate solution estimates while they are actually accurate enough for practical use. Another wrong conclusion could be that the PCG method applied to ssSNPBLUP poorly converges in comparison to ssGBLUP.

The two termination criteria CK and CM are related to the solutions of the system, and show similar patterns when applied to ssGBLUP and ssSNPBLUP, independently of the data set or of the preconditioner used. Because the number of SNP effects is a small proportion of the total number of equations in large evaluations, and because the SNP effect estimates are relatively small in comparison to the estimates of the other solutions, convergence properties of the PCG method applied to ssGBLUP and ssSNPBLUP based on CK and CM are comparable. Based on these observations and on the properties of the four different termination criteria, the termination criteria CK and CM can be recommended for comparing convergence properties of different models with similar vectors of solutions. Based on our results, it seems that the termination criterion CD can be a good alternative to CK and CM for comparing convergence properties when no implementation of these two latter criteria is available. Finally, the termination criteria CK and CM are also recommended for use in routine evaluations, because they allow the users to specify a desired relative accuracy in ${\mathbf {x}}$.

### Threshold for termination criteria

The threshold applied in this study for the termination criteria CK and CM was quite severe (i.e., $5*10^{-3}$). From a practical point of view, such an accuracy in the solutions is not required in routine evaluations, and the iterative process can be stopped sooner. For example, correlations between intermediate and true breeding values higher than 0.99990 were achieved within 27 and 77% of the total numbers of iterations needed to reach the severe threshold for CK and CM (Table 3). At this stage, Pearson correlations between intermediate and true SNP effects were all higher than 0.999. Based on the values obtained for the different termination criteria (Tables 2, 3), thresholds for both CK and CM around 0.2 for FIN, 2. for KAR, and 0.03 for LON and LON + block should therefore ensure enough accuracy in the solutions associated to the studied data sets for practical use. However, the adequacy of these thresholds should be checked and adapted for each evaluation.

### Implementation for CK and CM

In practice an estimate of the smallest active eigenvalue $\mu _{1}$ needed for the termination criterion CK, or of the effective spectral condition number needed for the termination criterion CM, is unknown until the termination of the iterative process. Our initial analyses with an approach based on the Lanczos method to update an estimate of $\mu _{1}$ at each iteration (similarly to the approach proposed by Kaasschieter [21]), showed us that such an approach may lead to stop the iterative process too soon, i.e. before a desired level of accuracy of the solutions is achieved (results not shown). This undesirable behavior was avoided by using conservative starting values for $\mu _{1}$ and $\kappa \left( \tilde{{\mathbf {M}}}^{-1}{\mathbf {C}} \right)$. These default values can be determined based on a previous analysis of a similar data set, or on previous experiences. Finally, the computation of the Ritz values at regular intervals (e.g., every 100-th iteration) allows to reduce its cost on the whole PCG process, even if this cost is relatively small. The main cost of the computation of the Ritz values is the eigendecomposition of a tridiagonal matrix of size equal to the number of iterations. In our analyses, each eigendecomposition took less than 1 s (wall clock time) until around 3000 iterations.

## Conclusions

In this study, we proposed two termination criteria for the PCG algorithm that consider the properties of the system of equations being solved, and that can be related to the relative error in the solutions. Based on our implemented approaches and results, we showed that the PCG algorithms applied to ssSNPBLUP and ssGBLUP show similar convergence patterns, provided that the termination criterion does not depend on the properties of the different systems, or considers them adequately. We also showed that the Ritz values, that are approximations of the eigenvalues of the preconditioned coefficient matrix and that can be computed directly from the PCG outputs, are a good tool to better understand the convergence behavior of the PCG algorithm.

## Supplementary Information

**Additional file 1.** Derivation of the termination criterion CR.

**Additional file 2.** Derivation of the termination criterion CM.

**Additional file 3.** Relationship between the residual of the system of equations of ssGBLUPand ssSNPBLUP.
