# Supplementary material for: Convergence behavior of single-step GBLUP and SNPBLUP for different termination criteria
Source: Genet Sel Evol. 2021 Apr 9;53:34. doi: 10.1186/s12711-021-00626-1 (PMC8034113; doi:10.1186/s12711-021-00626-1)
Supplement: Supplementary file 1 — Additional file 1. Derivation of the termination criterion CR. [file 12711_2021_626_MOESM1_ESM.pdf]

Additional file 1: Derivation of the termination criterion CR

The relative error in the solution $\mathbf{x}$ at the i-th iteration of the PCG method is defined as:

$$e_{r,i} = \frac{\|\mathbf{x} - \hat{\mathbf{x}}_i\|}{\|\mathbf{x}\|} \tag{1}$$

where $\hat{\mathbf{x}}_i$ is an approximate solution of $\mathbf{x}$ at the i-th iteration and $\|.\|$ is the 2-norm.

The system of linear equations has the form:

$$\mathbf{Cx} = \mathbf{b}$$

where $\mathbf{C}$ is a symmetric (semi-)definite coefficient matrix, and $\mathbf{b}$ is the right-hand side.

Using the fact that $\|\mathbf{b}\| = \|\mathbf{Cx}\| \leq \|\mathbf{C}\|\|\mathbf{x}\|$, it follows that:

$$\begin{aligned}
e_{r,i} &= \frac{\|\mathbf{x} - \hat{\mathbf{x}}_i\|}{\|\mathbf{x}\|} \\
&= \frac{\|\mathbf{C}^{-1}\left(\mathbf{b} - \mathbf{C}\hat{\mathbf{x}}_i\right)\|}{\|\mathbf{x}\|} \\
&= \frac{\|\mathbf{C}^{-1}\mathbf{r}_i\|}{\|\mathbf{x}\|} \\
&\leq \frac{\|\mathbf{C}^{-1}\|\|\mathbf{r}_i\|}{\|\mathbf{x}\|} \\
&\leq \|\mathbf{C}\|\|\mathbf{C}^{-1}\|\frac{\|\mathbf{r}_i\|}{\|\mathbf{b}\|} \\
&\leq \kappa\left(\mathbf{C}\right)\frac{\|\mathbf{r}_i\|}{\|\mathbf{b}\|}
\end{aligned}$$

where $\kappa\left(\mathbf{C}\right) = \|\mathbf{C}\|\|\mathbf{C}^{-1}\|$ is the effective spectral condition number of $\mathbf{C}$ defined as the ratio of its largest to smallest positive eigenvalues [20].
