# Supplementary material for: Convergence behavior of single-step GBLUP and SNPBLUP for different termination criteria
Source: Genet Sel Evol. 2021 Apr 9;53:34. doi: 10.1186/s12711-021-00626-1 (PMC8034113; doi:10.1186/s12711-021-00626-1)
Supplement: Supplementary file 2 — Additional file 2. Derivation of the termination criterion CM. [file 12711_2021_626_MOESM2_ESM.pdf]

Additional file 2: Derivation of the termination criterion CM

The relative error in the solution $\mathbf{x}$ at the i-th iteration of the PCG method is defined as:

$$e_{r,i} = \frac{\|\mathbf{x} - \hat{\mathbf{x}}_i\|}{\|\mathbf{x}\|} \tag{1}$$

where $\hat{\mathbf{x}}_i$ is an approximate solution of $\mathbf{x}$ at the i-th iteration and $\|.\|$ is the 2-norm.

The preconditioned system of linear equations has the form:

$$\tilde{\mathbf{M}}^{-1}\mathbf{C}\mathbf{x} = \tilde{\mathbf{M}}^{-1}\mathbf{b}$$

where $\mathbf{C}$ is a symmetric (semi-)definite coefficient matrix, $\mathbf{b}$ is the right-hand side, and $\tilde{\mathbf{M}}$ is a preconditioner.

Using the fact that $\|\tilde{\mathbf{M}}^{-1}\mathbf{b}\| = \|\tilde{\mathbf{M}}^{-1}\mathbf{C}\mathbf{x}\| \leq \|\tilde{\mathbf{M}}^{-1}\mathbf{C}\|\|\mathbf{x}\|$, it follows that:

$$\begin{aligned}
e_{r,i} &= \frac{\|\mathbf{x} - \hat{\mathbf{x}}_i\|}{\|\mathbf{x}\|} \\
&= \frac{\|\mathbf{C}^{-1}\left(\mathbf{b} - \mathbf{C}\hat{\mathbf{x}}_i\right)\|}{\|\mathbf{x}\|} \\
&= \frac{\|\mathbf{C}^{-1}\tilde{\mathbf{M}}\tilde{\mathbf{M}}^{-1}\mathbf{r}_i\|}{\|\mathbf{x}\|} \\
&\leq \frac{\|\left(\tilde{\mathbf{M}}^{-1}\mathbf{C}\right)^{-1}\|\|\tilde{\mathbf{M}}^{-1}\mathbf{r}_i\|}{\|\mathbf{x}\|} \\
&\leq \|\tilde{\mathbf{M}}^{-1}\mathbf{C}\|\|\left(\tilde{\mathbf{M}}^{-1}\mathbf{C}\right)^{-1}\|\frac{\|\tilde{\mathbf{M}}^{-1}\mathbf{r}_i\|}{\|\tilde{\mathbf{M}}^{-1}\mathbf{b}\|} \\
&\leq \kappa\left(\tilde{\mathbf{M}}^{-1}\mathbf{C}\right)\frac{\|\tilde{\mathbf{M}}^{-1}\mathbf{r}_i\|}{\|\tilde{\mathbf{M}}^{-1}\mathbf{b}\|}
\end{aligned}$$

where $\kappa\left(\tilde{\mathbf{M}}^{-1}\mathbf{C}\right) = \|\tilde{\mathbf{M}}^{-1}\mathbf{C}\|\|\left(\tilde{\mathbf{M}}^{-1}\mathbf{C}\right)^{-1}\|$ is the effective spectral condition number of $\tilde{\mathbf{M}}^{-1}\mathbf{C}$.
