# Supplementary material for: Convergence behavior of single-step GBLUP and SNPBLUP for different termination criteria
Source: Genet Sel Evol. 2021 Apr 9;53:34. doi: 10.1186/s12711-021-00626-1 (PMC8034113; doi:10.1186/s12711-021-00626-1)
Supplement: Supplementary file 3 — Additional file 3. Relationship between the residual of the system of equations of ssGBLUPand ssSNPBLUP. [file 12711_2021_626_MOESM3_ESM.pdf]

Additional file 3: Relationship between the residual of the system of equations of ssGBLUP and ssSNPBLUP

The system of equations for ssGBLUP can be written as:

$$\mathbf{C}_G\mathbf{x}_G = \mathbf{b}_G \tag{1}$$

where $\mathbf{C}_G$ is the symmetric (semi-)positive definite coefficient matrix, $\mathbf{x}_G$ is the vector of solutions, and $\mathbf{b}_G$ is the right-hand side associated with ssGBLUP.

Similarly, the system of equations for ssSNPBLUP proposed by Liu et al. [6] can be written as:

$$\mathbf{C}_L\mathbf{x}_L = \mathbf{b}_L \tag{2}$$

where $\mathbf{C}_L$ is the symmetric (semi-)positive definite coefficient matrix, $\mathbf{x}_L$ is the vector of solutions, and $\mathbf{b}_L$ is the right-hand side associated with ssSNPBLUP.

The system of equations (2) can be partitioned between the equations associated with the SNP effects (S) and the equations associated with the other effects (O), as follows:

$$\begin{bmatrix} \mathbf{C}_{L_{OO}} & \mathbf{C}_{L_{OS}} \\ \mathbf{C}'_{L_{OS}} & \mathbf{C}_{L_{SS}} \end{bmatrix} \begin{bmatrix} \mathbf{x}_{L_O} \\ \mathbf{x}_{L_S} \end{bmatrix} = \begin{bmatrix} \mathbf{b}_{L_O} \\ \mathbf{0} \end{bmatrix} \tag{3}$$

It can be shown that absorbing the equations corresponding to the SNP effects of the ssSNPBLUP system (3) leads to the ssGBLUP system (1), because $\mathbf{C}_G = \mathbf{C}_{L_{OO}} - \mathbf{C}_{L_{OS}}\mathbf{C}^{-1}_{L_{SS}}\mathbf{C}'_{L_{OS}}$, $\mathbf{x}_G = \mathbf{x}_{L_O}$, and $\mathbf{b}_G = \mathbf{b}_{L_O} - \mathbf{C}_{L_{OS}}\mathbf{C}^{-1}_{L_{SS}}\mathbf{0} = \mathbf{b}_{L_O}$. It is worth noting that $\mathbf{C}_G$ is the Schur complement of $\mathbf{C}_L$.

Both systems of equations (1) and (3) can be solved with the preconditioned conjugate gradient (PCG) method. For the following development, we will assume no rounding errors, such that an exact termination of the PCG method can occur. Furthermore, let's assume that the PCG method applied to the systems (1) and (3) results (in some state of their iterative process) in:

$$\hat{\mathbf{x}}_{G_i} = \hat{\mathbf{x}}_{L_{O_j}}$$

where $\hat{\mathbf{x}}_{G_i}$ is the vector of solutions obtained at the i-th iteration of the PCG method applied to the system (1), and $\hat{\mathbf{x}}_{L_{O_j}}$ is the vector of solutions obtained at the j-th iteration of the PCG method applied to the system (3).

Because the exact termination is not reached at the j-th iteration for the system (3), it follows that, at the j-th iteration:

$$\hat{\mathbf{x}}_{L_{S_j}} = -\mathbf{C}_{L_{SS}}^{-1}\mathbf{C}_{L_{OS}}^{'}\hat{\mathbf{x}}_{L_{O_j}} + \epsilon \tag{4}$$

Using Eq. (4), the 2-norm of the residual of the system of equations (3) at the j-th iteration, $\|\mathbf{r}_{L_j}\|$, is equal to:

$$\begin{aligned}
\|\mathbf{r}_{L_j}\| &= \left\| \begin{bmatrix} \mathbf{b}_{L_O} \\ \mathbf{0} \end{bmatrix} - \begin{bmatrix} \mathbf{C}_{L_{OO}} & \mathbf{C}_{L_{OS}} \\ \mathbf{C}_{L_{OS}}^{'} & \mathbf{C}_{L_{SS}} \end{bmatrix} \begin{bmatrix} \hat{\mathbf{x}}_{L_{O_j}} \\ \hat{\mathbf{x}}_{L_{S_j}} \end{bmatrix} \right\| \\
&= \left\| \begin{bmatrix} \mathbf{b}_{L_O} - \mathbf{C}_{L_{OO}}\hat{\mathbf{x}}_{L_{O_j}} - \mathbf{C}_{L_{OS}}\hat{\mathbf{x}}_{L_{S_j}} \\ -\mathbf{C}_{L_{OS}}^{'}\hat{\mathbf{x}}_{L_{O_j}} - \mathbf{C}_{L_{SS}}\hat{\mathbf{x}}_{L_{S_j}} \end{bmatrix} \right\| \\
&= \left\| \begin{bmatrix} \mathbf{b}_{L_O} - \mathbf{C}_{L_{OO}}\hat{\mathbf{x}}_{L_{O_j}} - \mathbf{C}_{L_{OS}}\left(-\mathbf{C}_{L_{SS}}^{-1}\mathbf{C}_{L_{OS}}^{'}\hat{\mathbf{x}}_{L_{O_j}} + \epsilon\right) \\ -\mathbf{C}_{L_{OS}}^{'}\hat{\mathbf{x}}_{L_{O_j}} - \mathbf{C}_{L_{SS}}\left(-\mathbf{C}_{L_{SS}}^{-1}\mathbf{C}_{L_{OS}}^{'}\hat{\mathbf{x}}_{L_{O_j}} + \epsilon\right) \end{bmatrix} \right\| \\
&= \left\| \begin{bmatrix} \mathbf{b}_{L_O} - \mathbf{C}_{L_{OO}}\hat{\mathbf{x}}_{L_{O_j}} + \mathbf{C}_{L_{OS}}\mathbf{C}_{L_{SS}}^{-1}\mathbf{C}_{L_{OS}}^{'}\hat{\mathbf{x}}_{L_{O_j}} - \mathbf{C}_{L_{OS}}\epsilon \\ -\mathbf{C}_{L_{OS}}^{'}\hat{\mathbf{x}}_{L_{O_j}} + \mathbf{C}_{L_{SS}}\mathbf{C}_{L_{SS}}^{-1}\mathbf{C}_{L_{OS}}^{'}\hat{\mathbf{x}}_{L_{O_j}} - \mathbf{C}_{L_{SS}}\epsilon \end{bmatrix} \right\| \\
&= \left\| \begin{bmatrix} \mathbf{b}_{L_O} - \left(\mathbf{C}_{L_{OO}} - \mathbf{C}_{L_{OS}}\mathbf{C}_{L_{SS}}^{-1}\mathbf{C}_{L_{OS}}^{'}\right)\hat{\mathbf{x}}_{L_{O_j}} - \mathbf{C}_{L_{OS}}\epsilon \\ -\mathbf{C}_{L_{SS}}\epsilon \end{bmatrix} \right\| \\
&= \left\| \begin{bmatrix} \mathbf{b}_{L_O} - \mathbf{C}_G\hat{\mathbf{x}}_{L_{O_j}} - \mathbf{C}_{L_{OS}}\epsilon \\ -\mathbf{C}_{L_{SS}}\epsilon \end{bmatrix} \right\| \\
&= \left\| \begin{bmatrix} \mathbf{r}_{G_i} - \mathbf{C}_{L_{OS}}\epsilon \\ -\mathbf{C}_{L_{SS}}\epsilon \end{bmatrix} \right\| \\
&= \left\| \begin{bmatrix} \mathbf{r}_{G_i} \\ \mathbf{0} \end{bmatrix} - \begin{bmatrix} \mathbf{C}_{L_{OS}}\epsilon \\ \mathbf{C}_{L_{SS}}\epsilon \end{bmatrix} \right\|
\end{aligned}$$

where $\mathbf{r}_{G_i}$ is the residual of the system (1) at the i-th iteration, and the 2-norm of an array $\mathbf{v}$ is defined as $\|\mathbf{v}\| = \sqrt{\sum_i (\mathbf{v}_i)^2}$.
